# Risk communication about work-related stress disorders in healthcare workers: a scoping review

**DOI:** 10.1007/s00420-022-01851-x

**Published:** 2022-03-16

**Authors:** Lima M. Emal, Sietske J. Tamminga, Joost G. Daams, Sanja Kezic, Danielle R. M. Timmermans, Frederieke G. Schaafsma, Henk F. van der Molen

**Affiliations:** 1 Department of Public and Occupational Health, Coronel Institute of Occupational Health, Amsterdam Public Health Research Institute, Amsterdam UMC, Universiteit van Amsterdam, Meibergdreef 9, PO Box 22700, 1100 DE Amsterdam, Noord-Holland The Netherlands; 2 Department of Public and Occupational Health, Amsterdam Public Health Research Institute, Amsterdam UMC, Vrije Universiteit Amsterdam, Amsterdam, Noord-Holland The Netherlands

**Keywords:** Health communication, Prevention, Risk factors, Trauma and stress-related disorders, Occupation disease, Healthcare workers

## Abstract

**Purposes:**

Healthcare workers are at risk of stress-related disorders. Risk communication can be an effective preventive health measure for some health risks, but is not yet common in the prevention of stress-related disorders in an occupational healthcare setting. The overall aim is to examine whether risk communication was part of interventions aimed at the prevention of stress-related disorders in healthcare workers.

**Method:**

We performed a scoping review using the framework of Arksey and O’Malley. We searched in Medline, Web of Science and PsychInfo for studies reporting on preventive interventions of stress-related disorders in healthcare workers between 2005 and December 2020. Studies were included when the intervention reported on at least one element of risk communication and one goal. We predefined four elements of risk communication: risk perception, communication of early stress symptoms, risk factors and prevention; and three goals: inform, stimulate informed decision-making and motivate action.

**Results:**

We included 23 studies that described 17 interventions. None of the included interventions were primarily developed as risk communication interventions, but all addressed the goals. Two interventions used all four elements of risk communication. The prominent mode of delivery was face to face, mostly delivered by researchers. Early stress symptoms and risk factors were measured by surveys.

**Conclusions:**

Risk communication on risk factors and early signs of stress-related disorders is not that well studied and evaluated in an occupational healthcare setting. Overall, the content of the communication was not based on the risk perception of the healthcare workers, which limited the likelihood of them taking action.

**Supplementary Information:**

The online version contains supplementary material available at 10.1007/s00420-022-01851-x.

## Introduction

The workplace is a key environment affecting the mental well-being of workers in that it provides financial security, time structure, social contacts, purpose, social identity and regular activity (Blake et al. 2020; Harnois 2000). On the other hand, unfavourable psychosocial work conditions may negatively influence mental well-being; in 2018 one in four workers in the European Union reported to be affected by work-related stress (Eurofound 2018). When work-related stress becomes excessive and coping strategies of the individual fail, it can result into a stress-related disorder (SRDs) such as anxiety, depression or burnout (Nieuwenhuijsen et al. 2010).

Within the working population, healthcare workers (HCWs) are significantly affected by SRDs, as studies report that up to 70% of various HCWs can suffer SRDs. During COVID-19, the prevalence of SRDs among HCWs has even further increased globally (Bridgeman et al. 2018; Salari et al. 2020). HCWs are affected by SRDs due to exposure to numerous risk factors related to the nature of their job, such as challenges of clinical work, time constraints, competing demands, scheduling, conflicting roles, effort–reward imbalance, high job demand, organizational injustice, lack of social support, high emotional demands and lack of decision authority (Bridgeman et al. 2018; van der Molen et al. 2020).

The effects of SRDs in HCWs can be more far reaching than in most other professions, as burnout may lead to an increase of medical errors and consequently affect patient safety and the quality of care (Shanafelt et al. 2010). Moreover, SRDs have adverse effects on the health of the HCWs (risk of coronary heart), organization (absenteeism, staff turnover) and society at large (economic losses). Because of the negative consequences of SRDs for the health and safety of HCWs and their patients, but also on organizations and society at large, prevention of adverse effects due to SRDs is important and has received a lot of attention in the past decade (Bartram et al. 2009; Bridgeman et al. 2018; Costello et al. 2019; Davey et al. 2009; De Hert 2020; Hassard et al. 2018; Li et al. 2015; Sara et al. 2018).

Communication plays an important role in health promotion; when it provides reliable and accurate information about health risks, it has the ability to enhance the knowledge and awareness of health risks. In turn, this can lead to behaviour that leads to health protection (Milne et al. 2000). Prevention of SRDs becomes easier when stress is still manageable, thus in its early stage, because serious consequences and risks can be reduced (Alberdi et al. 2016; Sharma et al. 2014). Communicating about (early) stress symptoms and risk factors to enhance knowledge and awareness of the risks may lead to HCWs protecting their health from health-threatening stress, possibly because HCWs can recognize the symptoms and risk factors timeously. Early symptoms of SRDs are gastrointestinal problems, sleep disturbances, dizziness, fatigue, eye strain, headache, loss of appetite and musculoskeletal pain (Sahlin et al. 2014). Thus, an intervention that detects and communicates about the (early) stress symptoms of SRDs and that identifies and communicates about underlying risk factors may prevent stress from becoming an SRD (Gartner et al. 2011; Sandler et al. 2010).

One approach to enhance knowledge and raise awareness of health risks of SRDs in HCWs is risk communication, as this provides accurate information about health risks. Providing accurate information may lead the individual to take informed decisions towards health risks (Claassen et al. 2016). Risk communication involves an accurate exchange of information about the existence, nature or severity of health risks and hazards and plays an important role in public health communication (DiClemente and Jackson 2017). The goals of risk communication are: (1) to inform, (2) to stimulate informed decision-making and/or (2) to motivate people towards action to protect health behaviours (Adil 2008; Diclemente and Jackson, 2017; Reynolds and Seegers 2005). The two goals—to stimulate informed decision-making and to motivate people towards action—are not mutually exclusive, because individuals who are well informed can make an informed decision not to take action. Because risk communication is rooted in various disciplines, there is no consensus on a common definition across and within disciplines (Löfstedt and Perri 2008). Therefore, based on the literature on risk communication from the public health arena, we conceptualized risk communication in an occupational healthcare setting as a dynamic, interactive dialogue and iterative process (Adil 2008; DiClemente and Jackson 2017; Nicholson 1999; Portell et al. 2014) between employers, workers and occupational health professionals embedded in a working context. In this dialogue, risk perception (Fischhoff et al. 1993; Slovic 1987) of occupational health professionals, employers, workers and other stakeholders is taken into account. The communication addresses the likelihood, severity and magnitude of potentially harmful health risks at work on the one hand and the possibilities for controlling or preventing work-related health risks on the other (Nicholson 1999). The goals of risk communication vary and can be distinguished as informing, stimulating informed decision-making, and actions to prevent work-related diseases (Reynolds and Seeger 2005).

From our conceptualization of risk communication, we defined four content elements and three goals of risk communication. The first conceptualized element of risk communication is risk perception. Risk perception involves people’s beliefs about and understanding of risks within their cultural and social context, including their previous experience and knowledge (Adil 2008; Fischhoff 2013; Morgan et al. 2001; Slovic 1987). Risk perception influences the likelihood of the recipient taking action towards prevention (Wachinger et al. 2013). For example, factors such as knowledge, experience, values, attitudes and emotions influence the thinking and judgement of individuals regarding the seriousness and acceptability of risks (Wachinger et al. 2013). People with low risk perception are less likely to respond to warnings or to take preventive measures than people with high risk perception (Ruin et al. 2007). Often, there is a discrepancy between the risk perception of experts and recipients of the risk communication, as experts may perceive risk as an epidemiological phenomenon, whereas recipients’ previous experience, attitudes and beliefs of the risk influence their risk perception (Hayenhjelm 2006; Wohlke et al. 2019). For example, recipients may be unable to understand a specific risk because scientific information is lacking or they do not understand this. Therefore, they attempt to make sense of the information based on what they already know, and often this is based on their intuition (Skarlatidou et al. 2012; Breakwell 2001). Hence, effective risk communication bridges the gap between the risk perception of recipients and that of experts and increases the chance of the recipient taking preventive action (Wachinger et al. 2013). A method for examining the risk perception of the experts and recipients is the mental model approach; this is a five-step approach that examines the cognitive beliefs of the risk communication recipients regarding the interpretation of health-risk messages (Morgan et al. 2001). With this approach, the gaps of knowledge about a particular disease or risk between the experts and recipients can be made clear by contrasting the two. The content and delivery of the risk communication matches the risk perception of recipients, so that the likelihood of risk communication being effective increases (Morgan et al. 2001). Besides (1) risk perception, communication about (2) (early) stress symptoms, (3) risk factors and (4) the possibilities for controlling or preventing health risks are distinguished. Risk communication provides a disclosure to a particular individual of the potential risks and benefits of taking action (Partridge 2017). Besides these four content elements of risk communication, three goals of risk communication are formulated: to (1) inform, (2) stimulate informed decision-making and (3) motivate towards action (Rowan 1991).

It is argued that people in general are not able to estimate health risks well. People tend to evaluate risks subjectively due to their own perception of certain health risks (Arezes and Miguel 2008). Therefore, providing risk communication is important as it takes people’s risk perception into account. When this is the case, risk communication has the ability to enhance people’s understanding of health threats, stimulate informed decision-making and also motivate them towards action to eliminate health risks (Freimuth and Quinn 2004; Lowbridge and Leask 2011). Examining and contrasting the mental models of the HCWs and experts regarding SRDs, with a mental model approach, allows accurate and relevant risk communication to be developed. For certain occupational hazards such as radiation or welding fumes, increasing awareness and communication of health risks has been evaluated and has proved to be effective as a preventive measure; this is also true for some risk communication studies on the prevention of cancer (Arezes and Miguel 2008; Cezar-Vaz et al. 2015; Partridge 2017; Schapira et al. 2006; Sheyn et al. 2008; Tilburt et al. 2011; Wohlke et al. 2019). As risk communication addresses all relevant causes, consequences and prevention of a particular risk or disease, timely risk communication about the risk factors, (early) stress symptoms and prevention may aid prevention of SRDs in the workplace as it enables individuals to make informed decisions and take action (Partridge, 2017). Hence, we hypothesize that this could also be effective as part of an intervention aimed at the prevention of SRDs in HCWs. However, this has not yet been evaluated or studied in terms of risk communication for early signs for SRDs in HCWs before. Therefore, we chose to conduct a scoping review, an approach that is often used when an area has not been reviewed comprehensively (Arksey and O'Malley 2005).

In this scoping review, we aim to examine whether risk communication was part of interventions aimed at the prevention of SRDs in HCWs. More specifically, the aims of this scoping review are to study: (1) to what extent are risk communication elements present in interventions that aim to prevent stress-related disorders in HCWs, (2) the content, mode of delivery and deliverer of these interventions, and (3) the assessment of early stress symptoms and risk factors in these interventions.

## Methods

The review has been performed according to the framework of Arksey and O’Malley (2005) and reported according to the checklist of Preferred Reporting Items for Systematic Reviews—extension for Scoping Reviews (Tricco et al. 2018).

### Information sources and search

In collaboration with a clinical librarian, the following three electronic databases were searched: Medline, Web of Science and PsychInfo. We included studies published between 2005 and December 2020. Initial Medline search results were analysed in VOS viewer to identify potentially irrelevant terms. Actual irrelevancy was checked before NOTing out in Medline and Embase to reduce recall noise and enhance precision of search results. The search strategy can be conceptually summarized as follows: (([occupational stress] AND [healthcare workers] AND [risk communication]) OR [relevant studies]) NOT [VOS terms]. The four predefined elements of risk communication (i.e. risk perception, communicating about (early) stress symptoms, communicating about risk factors, communicating about prevention) were part of the search. The electronic database search strategies used in this review can be found in Table 3 in the appendix. For the search of early stress symptoms, various terms were used (see Table 3 in the appendix).The protocol was not registered.

### Study selection

Studies that were published in scientific peer-reviewed journals in English or in Dutch were included in this review when they met the following criteria: i) description of an intervention that was aimed at preventing SRDs in HCWs, ii) included at least one of the four predefined elements of risk communication: (1) risk perception, (2) communicating about (early) stress symptoms, (3) communicating about risk factors, (4) communicating about prevention; and one of the three goals: (1) to inform, (2) to stimulate informed decision-making and (3) to motivate towards action. Systematic or scoping reviews and meta-analysis were excluded, but references of reviews were hand-screened for potentially relevant studies.

### Selection of sources of evidence

All of the retrieved studies were independently screened on the basis of title and abstract by at least two authors, against the above-mentioned specified inclusion and exclusion criteria to determine suitability for inclusion in the review. For screening of title and abstracts, we used the Web-based screening tool Rayyan (Ouzzani et al. 2016). One author (LE) screened all studies. Next, half of the studies were randomly assigned for screening to one author (ST) and the other half to other authors (SK, FS, HM). The selected studies were screened on full text, independently by two authors. Disagreements were resolved by discussion among the authors. Conflicts between the authors on whether to include or exclude studies on the basis of title and abstract were 3%.

### Data charting process

To gather the information required for data charting, a data-charting form was created. One author (LE) performed a pilot data extraction on a random sample of five studies and subsequently refined the form in consultation with the other authors. The author LE independently performed data extraction for all studies, which was then checked by ST. Disagreements between two authors were discussed to reach a final decision. Extracted data included publication characteristics such as author, year of publication, country, intervention receiver and intervention level. Additionally, we extracted which of the following four predefined elements of risk communication were used in the included interventions: (1) risk perception, (2) communication about risk factors, (3) communication about (early) symptoms, (4) communication about control/prevention, and which of the following three goals of risk communication were formulated in the studies: (1) purpose to inform, (2) stimulate informed decision-making and (3) motivate towards preventive action. We also extracted the mode of delivery of the included interventions, whether the intervention was delivered face to face, online, individual or in group form, and the intervention deliverer. Finally, to examine how the assessment of (early) stress symptoms and risk factors was done, we extracted measurement methods with reported cutoff points.

### Charting results

In the next phase we collated, summarized and reported the charted data from the data-extraction form. We reviewed the data-extraction chart referring to each study included to perform the research accurately and transparently. This content was reviewed by two research team members (LE and ST) to define the main trends and or commonalities and differences. To examine to what extent interventions or parts of interventions could be considered risk communication, we analysed whether the interventions contained the four elements and three goals of risk communication. To determine whether the content of the intervention was based on the risk perception, we examined whether the HCWs’ previous experience or (cultural) knowledge or attitudes or beliefs or values or emotions about (early) stress symptoms and risk factors of SRDs were enquired into and also taken into account in the intervention. If interventions communicated about physical and psychological symptoms of stress, we viewed that as communication about (early) stress symptoms. All communication about psychosocial risk factors for SRDs was reported as communication about risk factors. If the goal of the intervention was to reduce or prevent SRDs and the intervention communicated which preventive measures should be taken, we reported that as informing, stimulating informed decision-making and motivating towards action.

## Results

### Selected studies

**Fig. 1:**
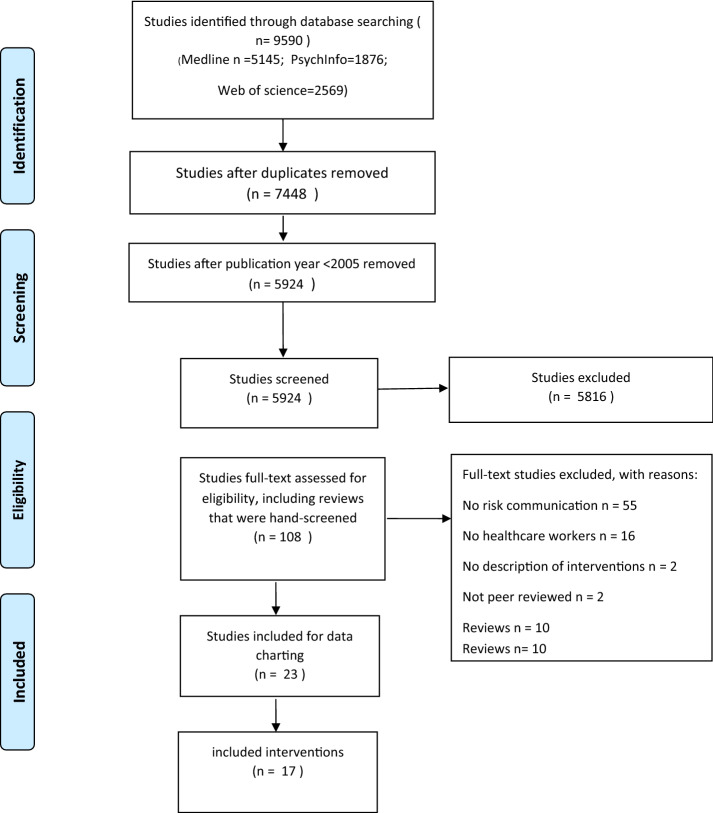
Prisma flow diagram of included studies

In the initial database search, 9595 studies were identified (Fig. 1). After removal of duplication, 7448 studies remained, and after deletion of studies that were published before 2005, 5924 studies were left for title and abstract screening. Eventually, 99 studies were assessed for eligibility at the level of full-text screening, and 10 reviews were hand-screened for relevant studies that had possibly been missed. After applying the inclusion and exclusion criteria, we retained a total of 23 studies (Fig. 1). During the full-text screening, studies were excluded when the intervention did not contain any form of communication about stress-related health risks (*n* = 55), the population were not HCWs (*n* = 16), there was no description of the intervention (*n* = 2), it was not peer-reviewed (*n* = 2) or the article was a review (*n* = 10).

### Study characteristics

Publication years ranged from 2007 to 2020. The majority of the studies were conducted in Western Europe, especially in the Netherlands (*n* = 12), followed by Italy (*n* = 3) (Table 1). In 17 studies, the intervention was at individual level. The setting in which the interventions were implemented was mostly hospitals (*n* = 17), mainly academic hospitals (Table 1 in the appendix).

**Table 1:** Study characteristics and intervention level of included studies (*n* = 23)

Authors/year of publication	Country	Study population	Intervention level
Arrigoni, et al. (2015)	Italy	Nurses	Individual
Blake et al. (2020)	UK	Various	Individual
d'Ettorre and Greco (2015)	Italy	Nurses and physicians	Organizational
Di Tecco et al. (2020)	Italy	Various	Both
Ericson-Lidman and Ahlin (2017)	Sweden	Nurses and managers	Individual
Gartner et al. (2011)	Netherlands	Nurses and various	Individual
Gartner et al. (2013)	Netherlands	Nurses and various	Individual
Havermans et al. (2018)	Netherlands	Various	Both
Isaksson Ro et al. (2010)	Norway	Physicians and various	Individual
Ketelaar et al. (2013a)	Netherlands	Nurses and various	Individual
Ketelaar et al. (2013b)	Netherlands	Nurses and various	Individual
Ketelaar et al. (2014a)	Netherlands	Nurses and various	Individual
Ketelaar et al. (2014b)	Netherlands	Nurses and various	Individual
Le Blanc et al. (2007)	Netherlands	Various	Organizational
Moll et al. (2015)	Canada	Various	Individual
Niks et al. (2013)	Netherlands	Various	Individual
Niks et al. (2018)	Netherlands	Various	Individual
Ruitenburg et al. (2015)	Netherlands	Physicians	Individual
Ruitenburg et al. (2016)	Netherlands	Physicians	Both
Schneider et al. (2019)	Germany	Nurses and physicians	Individual
Shanafelt et al. (2014)	United States of America	Physicians	Individual
Uchiyama et al. (2013)	Japan	Nurses	Both
Weiner et al. (2020)	France	Various	Individual

### Presence of the content elements of risk communication, risk perception, early stress symptoms, risk factors and prevention

The studies that were included counted 17 different interventions with none of them primarily aimed at and developed as risk communication (Table 2 and Table 2 in the appendix). Two interventions (Arrigoni et al. 2015; Ericson-Lidman and Ahlin 2017) contained all four predefined content elements and three goals of risk communication. That is, in the intervention of Arrigoni et al. (2015), risk perception of the experts and HCWs was taken into account, since the content of the educational plan was developed via a two-way dialogue between the researchers and the nurses. The exercises in the educational plan were based on real cases brought in by the nurses themselves. Arrigoni et al. (2015) communicated about (early) stress symptoms such as emotional distress and risk factors such as lack of social support and stressful work situations. Communication about prevention focused on coping with stress. The second intervention that contained all predefined elements and goals of risk communication is the study by Ericson-Lidman and Ahlin (2017). The content of the intervention was created by taking the risk perception of the HCWs into account, asking the HCWs to review their knowledge about factors that contributed to an increase of their troubled conscience. The HCWs then defined the specific gaps in knowledge that they found to be relevant to the researchers. Then, during interviews, the researchers and HCWs discussed, identified, prioritized and brainstormed about the situations that caused troubled conscience at work. After this risk perception analysis, the content of the communication was about (early) stress symptoms such as behavioural symptoms and risk factors regarding deficient teams and non-functional mealtime schedule. HCWs themselves decided which preventive actions they would take.

**Table 2:** Overview of elements and goals of risk communication in included interventions

Authors/year of publication	Elements of risk communication				Goals risk communication		
	Risk perception	Communication about: (early) stress symptoms	Communication about: risk factors	Communication about: prevention	Informing	Stimulating informed decision-making	Taking action
Arrigoni et al. (2015)	+	+	+	+	+	+	+
Blake et al. (2020)	−	+	+	+	+	+	+
d'Ettorre and Greco (2015)	−	−	+	+	+	+	+
Di Tecco et al. (2020)	+	−	+	+	+	+	+
Ericson-Lidman and Ahlin (2017)	+	+	+	+	+	+	+
Gartner et al. (2011, 2013) (Ketelaar et al. 2013b, 2014a)	−	+	+	+	+	+	+
Havermans et al. (2018)	−	+	+	+	+	+	+
Isaksson Ro et al. (2010)	+	+	−	+	+	+	+
Ketelaar et al. (2013b, 2014a)	−	+	+	+	+	+	+
Le Blanc et al. (2007)	+	−	+	+	+	+	+
Moll et al. (2015)	−	+	−	+	+	+	+
Niks et al. (2013, 2018)	−	+	+	+	+	+	+
Ruitenburg et al. (2015, 2016)	−	+	+	+	+	+	+
Schneider et al. (2019)	−	+	+	+	+	+	+
Shanafelt et al. (2014)	−	+	+	+	+	+	+
Uchiyama et al. (2013)	−	+	+	+	+	+	+
Weiner et al. (2020)	−	+	+	+	+	+	+

Three interventions (Di Tecco et al. 2020; Isaksson Ro et al. 2010; Le Blanc et al. 2007) developed the content of their intervention by taking the risk perception of both intervention developers and recipients into account, but did not communicate about (early) stress symptoms. In the intervention of Di Tecco et al. (2020), the researchers conducted an assessment based on the HCWs’ perception of psychosocial risks. The communication about risk factors included working conditions and job satisfaction. Together with other stakeholders, HCWs discussed which preventive measures were appropriate. Content of the communication of Isaksson Ro et al. (2010) was based on the work-related and private contextual factors contributing to stress according to HCWs themselves. Their goal was to reduce emotional exhaustion and job stress and increase emotion-focused coping. Le Blanc et al. (2007) communicated about risk factors by educating HCWs about social support networks and balancing job-related investments and outcomes. Risk perception was taken into account by discussing the HCWs’ perception of the main sources of job stress. This was performed through interviews, where the HCWs’ local context was taken into account. For prevention, a plan for taking action to cope with most stressors was discussed.

The communication of the following 12 interventions did not take the risk perception of the recipients into account. In the intervention of Gartner, Ketelaar and Ruitenberg’s communication focused on mental health complaints and work functioning impairments, psychological health complaints and work ability. Furthermore, the communication addressed prevention by giving advice and a consultation with an occupational physician (Gartner et al. 2011, 2013; Ketelaar et al. 2013a, b, 2014a, b; Ruitenburg et al. 2015, 2016).

Blake et al. (2020), d'Ettorre and Greco (2015), Uchiyama et al. (2013) and Havermans et al. (2018) communicated about risk factors and (early) symptoms of stress. Moll et al. (2015) communicated about mental health literacy and Shanafelt et al. (2014) provided individualized feedback on HCWs’ well-being in relation to risk of fatigue, career satisfaction, meaning in work, risk of suicidal ideation, risk degree of distress and mental quality of life. In terms of prevention, these interventions provide Web links and phone numbers of different institutions.

Niks et al. (2013, 2018) communicated the results of a survey about psychosocial diagnoses, Schneider et al. (2019) communicated work system factors and mental well-being and the Weiner et al.’s (2020) study communicated about the psychological mechanism of stress. In the interventions of Niks et al. (2013) and Schneider et al. (2019), preventive measures were not explicitly communicated, but HCWs discussed potential solutions and defined a plan with concrete measures, while the communication of Weiner et al. (2020) about prevention referred to useful behaviour during stressful situations.

### Presence of the goals of risk communication, informing, stimulating informed decision-making and motivating actions

All 17 interventions contained all three goals of risk communication, i.e. informing, stimulating informed decision-making and motivating towards action. The primary goal of all 17 interventions was to reduce or prevent SRDs and thus to change behaviour. Informing HCWs about SRDs was part of the intervention goal; some studies gave information during training (Arrigoni et al. 2015), digitally (Blake et al. 2020; Ketelaar et al. 2013a, b; Shanafelt et al. 2014; Weiner et al. 2020), during discussion sessions (d'Ettorre and Greco 2015; Di Tecco et al. 2020; Niks et al. 2013, 2018; Uchiyama et al. 2013) or by the occupational physician (Gartner et al. 2011, 2013; Ketelaar et al. 2013a, b, 2014a, b; Ruitenburg et al. 2015, 2016). Through the giving of information, in all interventions HCWs were encouraged to take action to protect their health, but they could decide this for themselves, or they could decide not to do anything about it.

### Mode of delivery of the interventions

Table 3 summarizes the mode of delivery of the interventions. In 13 interventions (Arrigoni et al. 2015; d'Ettorre and Greco 2015; Di Tecco et al. 2020; Ericson-Lidman and Ahlin 2017; Isaksson Ro et al. 2010; Le Blanc et al. 2007; Niks et al. 2013, 2018, 2016; Ruitenburg et al. 2015; Schneider et al. 2019; Uchiyama et al. 2013; Weltermann et al. 2020), the mode of delivery was face to face, in three (Blake et al. 2020; Ketelaar et al. 2013a, b, 2014a, b) it was online and in two (Gartner et al. 2011, 2013; Havermans et al. 2018; Ketelaar et al. 2013a, b, 2014a, b; Slater et al. 2018; Weiner et al. 2020) blended. The intervention deliverer varied between the studies. In seven studies, intervention deliverers were the researchers of the study (Arrigoni et al. 2015; d'Ettorre and Greco 2015; Di Tecco et al. 2020; Ketelaar et al. 2013a, b, 2014a, b), and in six interventions (Di Tecco et al. 2020; Gartner et al. 2011, 2013, 2016; Ketelaar et al. 2013a, b, 2014a, b; Le Blanc et al. 2007; Ruitenburg et al. 2015; Weiner et al. 2020) these were various HCWs. In the other two interventions, there was a mix of deliverers of team counsellors together with the peer educators or researchers (Le Blanc et al. 2007; Moll et al. 2015). Five interventions were in group settings (Arrigoni et al. 2015; Le Blanc et al. 2007; Moll et al. 2015; Niks et al. 2013, 2018; Uchiyama et al. 2013), six individual (Blake et al. 2020; Ketelaar et al. 2013a, b; Ruitenburg et al. 2015, 2016; Schneider et al. 2019; Shanafelt et al. 2014; Weiner et al. 2020) and six both individual and in group setting (d'Ettorre and Greco 2015; Di Tecco et al. 2020; Ericson-Lidman and Ahlin 2017; Havermans et al. 2018; Isaksson Ro et al. 2010).

**Table 3:** Overview of the mode of delivery and deliverer of the interventions

Author(s)/year	Mode of delivery of the intervention			Intervention deliverer
	Face-to-face	Online	Individual or group	
Arrigoni et al. (2015)	YES	NO	Group	Researcher
Blake et al. (2020)	NO	YES	Individual	Researcher
d'Ettorre and Greco (2015)	YES	NO	Both	Researcher
Di Tecco et al. (2020)	YES	NO	Both	Occupational safety professionals, employees and managers
Ericson-Lidman and Ahlin (2017)	YES	NO	Both	Researcher
Gartner et al. (2011, 2013) Ketelaar et al. (2013b, 2014a)	YES	YES	Individual	Occupational physicians and researchers
Havermans et al. (2018)	YES	YES	Both	NR
Isaksson Ro et al. (2010)	YES	NO	Both	Psychiatrist, counsellors and specialist in occupational medicine, occupational therapist
Ketelaar et al. (2013b, 2014a)	NO	YES	Both	Researchers
Le Blanc et al. (2007)	YES	NO	Group	Registered behaviour therapists
Moll et al. (2015)	YES	YES	Group	Researchers and trained peer educators who have personal experience
Niks et al. (2013, 2018)	YES	NA	Group	Researchers, management
Ruitenburg et al. (2015, 2016)	YES	NO	Individual	Occupational physicians
Schneider et al. (2019)	YES	NO	Individual	Researchers
Shanafelt et al. (2014)	NO	YES	Individual	NR
Uchiyama et al. (2013)	YES	NO	Group	Researchers and nurses
Weiner et al. (2020)	NO	YES	Individual	Psychologist

*NR* not reported

### Assessment of early stress symptoms, risk factors and prevention

Table 4 summarizes how early stress symptoms and risk factors were measured in the intervention studies. Ten interventions (Arrigoni et al. 2015; d'Ettorre and Greco 2015; Ericson-Lidman and Ahlin 2017; Gartner et al. 2011, 2013, 2016, 2018; Ketelaar et al. 2013a, b, 2014a, b; Niks et al. 2013; Ruitenburg et al. 2015; Schneider et al. 2019; Shanafelt et al. 2014; Weiner et al. 2020) used questionnaires to detect signs of work-related stress and reported cutoff points were used to detect HCWs who were at risk. The (early) signs that were measured varied from stress (Arrigoni et al. 2015; Blake et al. 2020; Havermans et al. 2018; Weiner et al. 2020), behavioural symptoms (Ericson-Lidman and Ahlin 2017), impaired mental health (Gartner et al. 2011, 2013; Ketelaar et al. 2013a, b, 2014a, b), burnout/perceived stress (Isaksson Ro et al. 2010), mental health literacy (Moll et al. 2015), emotional physical recovery (Niks et al. 2013, 2018), psychological health complaints (Ruitenburg et al. 2015, 2016), mental well-being (Schneider et al. 2019; Uchiyama et al. 2013) to personal well-being (Shanafelt et al. 2014). Measured risk factors were (unspecified) psychosocial risk factors (Blake et al. 2020; d'Ettorre and Greco, 2015; Havermans et al. 2018; Ruitenburg et al. 2015, 2016; Schneider et al. 2019; Uchiyama et al. 2013; Weiner et al. 2020), lack of social support (Arrigoni et al. 2015), working conditions and job satisfaction (Di Tecco et al. 2020), deficient teams and non-functional mealtime schedule (Ericson-Lidman and Ahlin, 2017), impaired work functioning (Gartner et al. 2011, 2013; Ketelaar et al. 2013a, b, 2014a, b), support network and job-related investments (Le Blanc et al. 2007), job demands and job resources (Niks et al. 2013, 2018) and career satisfaction and meaning of work (Shanafelt et al. 2014). In 15 interventions (Arrigoni et al. 2015; Blake et al. 2020; Ericson-Lidman and Ahlin 2017; Gartner et al. 2011, 2013, 2016, 2018; Havermans et al. 2018; Isaksson Ro et al. 2010; Ketelaar et al. 2013a, b, 2014a, b; Le Blanc et al. 2007; Moll et al. 2015; Niks et al. 2013; Ruitenburg et al. 2015; Schneider et al. 2019; Shanafelt et al. 2014; Uchiyama et al. 2013; Weiner et al. 2020), the questionnaires were self-administered, in 1 it was conducted by interviews (d'Ettorre and Greco 2015) and in another one also accompanied with objective data (Di Tecco et al. 2020).

**Table 4:** Stress symptoms and risk factors that were measured and communicated

Author(s)/year	(Early) stress symptoms	Risk factors	Measurement methods		Assessment of (early) stress symptoms and risk factors	Cut-off-point reported	
			(Early) stress symptoms	Risk factors		(Early) stress symptoms	Risk factors
Arrigoni et al. (2015)	Stress symptoms	Lack of social support, work situations	BPI	HPSCS	Self-Administrated	YES	YES
Blake et al. (2020)	Stress symptoms	Psychosocial risk factors	NO	NO	NO	NO	NO
d'Ettorre and Greco (2015)	NO	Psychosocial risk factors	NO	Multidimensional validated tool	Interviews	NO	YES
Di Tecco et al. (2020)	NO	Working conditions, job satisfaction	NO	I-Check and Management Standards Indicator Tool	Objective data and self-administrated	NO	NO
Ericson-Lidman and Ahlin (2017)	Behavioural symptoms	Deficient teams, non-functional mealtime schedule	NO	NO	Self-administrated	YES	YES
Gartner et al. (2011, 2013) Ketelaar et al. (2013b, 2014a)	Impaired mental health	Impaired work functioning	4DSQ BSI PHQ-15 SVL One item: “Do you think that your work has negative consequences for your mental health?”	NWFQ	Self-administrated	YES	YES
Havermans et al. (2018)	Stress symptoms	Psychosocial risk factors	NO	NO	Self-administrated	NO	NO
Isaksson Ro et al. (2010)	Burnout, perceived stress	NO	NO	NO	Self-administrated	NO	NO
Ketelaar et al. (2013b, 2014a)	Impaired mental health	Impaired work functioning	4DSQ	QEEW NWFQ	Self-administrated	YES	YES
Le Blanc et al. (2007)	NO	Support network,job-related investments	NO	NO	Self-administrated	NO	NO
Moll et al. (2015)	Mental health literacy	NO	NO	NO	Self-administrated	NO	NO
Niks et al. (2013, 2018)	Emotional, physical recovery	Job demands,Job resources	NO	NO	Self-administrated	YES	YES
Ruitenburg et al. (2015, 2016)	Psychological health complaints	Psychosocial risk factors	Job specific Worker’surveillance	Job specific worker’s health surveillance	Self-administrated	YES	YES
Schneider et al. (2019)	Mental well-being	Work system factors	NO	NO	Self-administrated	YES	YES
Shanafelt et al. (2014)	Personal well-being	Career satisfaction, meaning in work	MPWBI	MPWBI	Self-administrated	YES	YES
Uchiyama et al. (2013)	Mental health	Psychosocial risk factors	NO	NO	Self-Administrated	NO	NO
Weiner et al. (2020)	Stress	Psychosocial risk factors	NO	PHQ2, SF-PCL-5, CD-RISC 2, ISI, ARQ, CEQ, CSQ-8	Self-administrated	NO	YES

*BPI* Burnout Potential Inventory, *HSPSCS* The Health Profession Stress and Coping Scale, *4DSQ* Four-Dimensional Symptoms Questionnaire, *BSI* Brief Symptom Inventory, *PHQ-15* Patient HealthQuestionnaire, *SVL* the Schok Verwerkings Lijst, *NWFQ* Nurse work functioning questionnaire, *QEEW* Questionnaire on the Experience and Evaluation of Work, *MPWBI* Mayo Clinic Physician Well-Being Index, *PHQ2* the Patient Health Questionnaire—2 items version, *SF-PCL-5* the Short Form Posttraumatic Stress Disorder Checklist 5, *CD-RISC 2* the Connor–Davidson Resilience Scale—2 items version, *ISI* Insomnia Severity Index, *ARQ* the Affective Rumination Questionnaire, *CEQ* Credibility and Expectancy Questionnaire

## Discussion

### Main findings and interpretation

In this scoping review, we examined whether risk communication was part of interventions aimed at the prevention of SRDs in HCWs, based on the assumption that communicating health risks has the potential to enhance knowledge and awareness about health risks and might therefore be a promising approach to activate towards action. More specifically, we examined which risk communication elements were present in these interventions and collated the content and mode of delivery of these interventions. We did not find any intervention that was primarily developed as risk communication. However, two interventions contained all predefined elements and goals of risk communication. However, these two interventions did not explicitly name risk communication nor were they intended to be a risk communication health measure (Arrigoni et al. 2015; Blake et al. 2020; Ericson-Lidman and Ahlin 2017). All of the included studies contained all three goals of risk communication. This is not surprising given that these studies were developed as behaviour change interventions and not as risk communications per se.

Our results suggest that risk communication is not that well studied and evaluated in occupational healthcare when it comes to preventing SRDs for HCWs. Considering the importance and consequences of SRDs in HCWs and also how often communicating and raising awareness of health risks as a preventive measure has proven to be effective (Partridge 2017; Rainey et al. 2020; Schapira et al. 2006; Tilburt et al. 2011; Wohlke et al. 2019), it is worth investigating whether risk communication is a promising approach in the occupational healthcare setting for SRDs in HCWs. Developing and applying risk communication about SRDs in HCWs can be complex. To reduce or prevent SRDs, a certain behaviour is required from the HCWs; however, it is known that promoting work safety behaviour cannot be achieved by just communicating (Fischhoff 2012). The work environment is important; it must be set up in such a way that it is safe (Stege et al. 2021). For example, many risk factors for SRDs are organizational and cannot be changed by HCWs themselves alone. In this matter, an occupational physician or other health professionals can mediate between the HCWs and the employer.

It is worth noting that five out of the 17 interventions (Di Tecco et al. 2020; Havermans et al. 2018; Isaksson Ro et al. 2010; Le Blanc et al. 2007; Moll et al. 2015; Slater et al. 2018; Weltermann et al. 2020) missed communication of (early) stress symptoms and risk factors as a crucial element of risk communication. To achieve its goal, risk communication should be complete and should communicate about the risk of the disease and provide clear disclosure of the potential risks and benefits of a given intervention for the individual (Partridge 2017). According to the common-sense model, symptoms, consequences, causes and controllability of a health threat are influenced by illness perceptions (Leventhal et al. 2016). This illness perception influences coping strategies, which in turn influence outcomes. Thus, communicating about all possible risks such as (early) symptoms, risk factors and also which preventive actions could be taken is important for health protection (Milne et al. 2000).

Ten interventions (Gartner et al. 2011, 2013, 2016, 2018; Ketelaar et al. 2013a, b, 2014a, b; Le Blanc et al. 2007; Niks et al. 2013; Ruitenburg et al. 2015; Uchiyama et al. 2013) contained all of the predefined elements of risk communication and goals except for risk perception. The studies (Arrigoni et al. 2015; Blake et al. 2020; Di Tecco et al. 2020; Ericson-Lidman and Ahlin 2017; Isaksson Ro et al. 2010; Le Blanc et al. 2007) that included risk perception of the HCWs about SRDs assessed the risk perception by asking the HCWs about the factors that influence their risk perception of SRDs and included the risk perception in the communication. However, including the risk perception in the communication was done through interviews and not through a mental model approach. The mental model approach seeks to construct the mental model of scientific experts and recipients with regard to risks (Morgan et al. 2001). By contrasting the mental model of the scientific experts with that of the HCWs, it is possible to identify the specific information needs: the gaps in knowledge about causes, consequences and prevention of SRDs in HCWs (Morgan et al. 2001). It is important to take the risk perception of recipients into account because neglecting the risk perception of the HCWs in the development of the risk communication may affect the willingness of HCWs to take preventive action (Wachinger et al. 2013). It can be hypothesized that HCWs not taking action towards prevention is due the discrepancy between the mental models of the intervention deliverers and the HCWs, as it is known that HCWs often perceive severe stress as part of their job (Shanafelt et al. 2014). Therefore, studies that successfully develop the content of their health communication based on the mental model approach and so engage risk perception of the recipients in their intervention will increase the likelihood that healthy behaviours are adopted (Ferrer and Klein 2015).

The intervention deliverers in the included studies were mostly researchers or other HCWs. This can be a disadvantage for two reasons. First, an important factor for effective risk communication is the trustworthiness and credibility of the deliverer (Trettin and Musham 2000; Wachinger et al. 2013). Trusting the deliverer increases the chances of someone taking action towards prevention the risks (Trettin and Musham 2000; Wachinger et al. 2013). Second, it is unclear what happened with the intervention after the study had ended, and the researchers were no longer available to deliver the risk communication.

Finally, all studies measured (early) stress symptoms and risk factors through self-reporting, mainly by surveys. However, there are several limitations with surveys, a recent systematic review concerning occupational burnout concluded that the measurement quality of these surveys is often not adequate (Shoman et al. 2021). In a dialogue the supervisor can also detect early stress symptoms, the supervisor can find out whether and how much stress the HCW experiences (Bakhuys et al. 2020). Nonetheless, this can be challenging in practice as supervisors have reported difficulty in detecting early stress symptoms of their employees especially when the person concerned performed well and appeared happy (Eskilsson et al. 2021). An additional way of early detection of stress is objective assessment of stress symptoms, e.g. by measuring stress-associated biomarkers (Kaczor et al. 2020). A recent pilot study has shown that biomarkers detected by wearable sensor might be useful to identify HCWs stress in the clinical environment. An advantage of measuring stress-associated biomarkers is that it detects stress before it is reported or even recognized by the individual. The first signs of stress are already measurable and this can contribute to managing the stress-levels, especially in HCWs who have a higher risk for SRDs (Kaczor et al. 2020).

### Strength and limitations

A strength of this scoping review is that we have systematically investigated risk communication on risk factors and early signs of SRDs in HCWs, because some forms of raising awareness and communicating health risks as a preventive measure have been proven to be effective in public health (Arezes and Miguel 2008; Cezar-Vaz et al. 2015; Partridge 2017; Schapira et al. 2006; Sheyn et al. 2008; Tilburt et al. 2011; Wohlke et al. 2019), but not yet common in the prevention of SRDs in an occupational healthcare setting. Also, the search was conducted by an experienced librarian and included a comprehensive literature search. Another strength is that our conceptualization of risk communication is based on broadly published public health risk communication literature (DiClemente and Jackson 2017; Fischhoff 2013; Nicholson 1999; Portell et al. 2014; Slovic 1987), which makes our conceptualization more complete. A limitation of this review is that the search strategy was limited to publications in English, meaning that potentially relevant studies in other languages were missed. Also, the criteria on which we decided whether risk perception was included in the communication might be considered a limitation, because our criteria were rather broad. That is, when HCWs mentioned factors that influenced their risk perception (e.g. cultural knowledge, attitudes, beliefs, values or emotions) and these factors were taken into account in the communication, we considered it risk perception. However, this is not in line with the risk communication literature, in which risk perception should be based on the mental model approach (Morgan et al. 2001). Nonetheless, this approach suited the broad research question of this scoping review. Another limitation is that, for our assessment of which goals of risk communication were part of the intervention, we grouped the goals together. When the main objective of the intervention was to prevent or reduce SRDs we characterized it as motivating towards action. If the intervention communicated about (early) stress symptoms and risk factors for SRDs, we assessed that as informing. If these two goals were included, we then characterized the goal of the interventions as stimulating informed decisions, because HCWs are informed about which actions to take, but do not have to. This is not in line with the goals in risk communication literature, as the goals are distinctive from each other. Nonetheless, the studies we included intended to prevent or reduce SRDs; when it comes to SRDs, only informing is not sufficient. Stimulating informed decision-making and motivating towards action are more important for health protection. Thus in most behavioural change interventions there is less differentiation between the three goals of risk communication.

### Recommendations for further research and practice

HCWs are significantly affected by SRDs and this has far-reaching consequences for the overall health of the HCWs and also for the healthcare sector (Bridgeman et al. 2018; Davey et al. 2009; Li et al. 2015; Shanafelt et al. 2010; West et al. 2006). Many interventions have been developed to prevent SRDs in HCWs, but have not always been effective (Aryankhesal et al. 2019). We recommend that interventions that are aimed to prevent SRDs in HCWs also develop risk communication as part of their intervention to enhance the likelihood of HCWs taking preventive actions. Furthermore, to develop risk communication specifically of early signs of SRDs in HCWs, we recommend that more research be conducted on the risk perception of the HCWs, since identifying the factors that influence risk perception is essential to our understanding of risk structure and formation (Yang 2014). Also, to develop risk communication the mental model approach should be used, to contrast the mental models of the HCWs and other stakeholders, so that the gaps in knowledge and information needs is researched. Furthermore, it is important to develop and offer interventions that also target work-related risk factors. In practice this means making adjustments to the most important risk factors that are associated with SRDs. A combination of interventions at the organizational-level and individual level have been proven to be more effective than interventions that are only aimed at individual level, this combination seems more effective and sustainable (Lamontagne et al. 2007; Uchiyama et al. 2013). Finally, the various methods to detect stress have their own limitations (Eskilsson et al. 2021; Shoman et al. 2021); therefore, another way to detect stress might be the use of valid biomarkers (Noushad et al. 2021). However, the measurement techniques mentioned do not have to be mutually exclusive, but could complement each other.

## Conclusion

We conclude that risk communication is not that well studied and evaluated in an occupational healthcare setting when it comes to preventing SRDs in HCWs. The effectiveness of interventions that were aimed to prevent or reduce SRDs in HCWs would potentially increase if risk communication were to be part of broader and multifaceted preventive interventions. Overall, most interventions we reviewed did not explicitly measure the risk perception of the HCWs regarding SRDs, which is thought to be an important element of risk communication because it increases the likelihood for HCWs to take actions to protect their (mental) health. All interventions contained the three goals of risk communication. The mode of delivery of the interventions were mostly face-2-face, at individual level and in a group setting. The intervention deliverer were researchers or HCWs. Early stress symptoms and risk factors were measured by self-reported surveys.

## Supplementary Information

Below is the link to the electronic supplementary material.

Supplementary file1 (DOCX 50 kb)

Supplementary file2 (DOCX 53 kb)

Supplementary file3 (DOCX 17 kb)

## Declarations

### Approval of the research protocol

N/A.

### Registry and the registration no. of the study/trial

N/A.

### Animal studies

N/A.
