# Supplementary material for: Risk communication about work-related stress disorders in healthcare workers: a scoping review
Source: Int Arch Occup Environ Health. 2022 Mar 16;95(6):1195–208. doi: 10.1007/s00420-022-01851-x (PMC8923828; doi:10.1007/s00420-022-01851-x)
Supplement: Supplementary file 1 — Supplementary file1 (DOCX 50 kb) [file 420_2022_1851_MOESM1_ESM.docx]

Appendix 1.

| Authors/ year of publication & country | Total sample size | Outcome | Setting |
| --- | --- | --- | --- |
|  |  |  |  |
| Arrigoni, et al. 2015^1^ | n=511 | Coping mechanism  Stress domains | Three hospitals |
| Blake et al. 2020^2^ | n=107 | nr | Healthcare organizations |
| D'ettore et al. 2014^3^ | NR | Sentinel events, work content factors | Hospital departments and care services of health district |
| Di Tecco et al. 2020^4^ | NR | Psychosocial risks, Job satisfaction. | Two large hospitals |
| Ericson-Lidman et al. 2017^5^ | n=76 | Stress of conscience | Residential care facilities for older adults in a medium-size community |
| Gartner et al. 2011^6^ | n=1731 | Help-seeking behavior Mental health complaints Absenteeism, Work functioning  Help-seeking behavior, Work ability, Turnover intention, Wellbeing Work productivity | Dutch Academic Medical Center |
| Gartner et al. 2013^7^ | n= 379 | Help-seeking behavior Mental health complaints Absenteeism, Work functioning  Help-seeking behavior, Work ability, Turnover intention, Wellbeing Work productivity | Dutch Academic Medical Center |
| Havermans et al. 2021^8^ | n= 304 | Help-Seeking Behavior, Impaired Work functioning, Mental Health Complaints  Risky Drinking Behavior Depression Anxiety Posttraumatic Stress Disorder | Large Dutch healthcare organization |
| Isaksson et al. 2010^9^ | n= 184 | Stress Psychological demands, Social support Autonomy, Level of implementation. | The Resource Centre |
| Ketelaar et al. 2013^10^ | n=369 | Help-seeking behavior Mental health complaints Absenteeism, Work functioning  Help-seeking behavior, Work ability, Turnover intention, Wellbeing Work productivity | Academic medical center |
| Ketelaar et al. 2013^11^ | n= 1140 | Help-seeking behavior Mental health complaints Absenteeism, Work functioning  Help-seeking behavior, Work ability, Turnover intention, Wellbeing Work productivity | Academic medical center in the Netherlands |
| Ketelaar et al. 2014^12^ | n=128 | Help-seeking behavior Mental health complaints Absenteeism, Work functioning  Help-seeking behavior, Work ability, Turnover intention, Wellbeing Work productivity | Academic medical center in the Netherlands |
| Ketelaar et al. 2014^13^ | n=1170 | Help-seeking behavior Mental health complaints Absenteeism, Work functioning  Help-seeking behavior, Work ability, Turnover intention, Wellbeing Work productivity | Academic medical center in the Netherlands |
| Le Blanc et al. 2007^14^ | n= 664 | Burn-out  Social support Participation in decision making  Job control  Job demands | 18 general hospitals spread throughout the Netherlands. |
| Moll et al. 2015^15^ | NA | Mental health literacy Attitudes towards Mental illness help seeking  Outreach behavior | Large healthcare facility & mid-sized hospital. |
| Niks et al. 2013^16^ | NA | Job demands, Job resource, Recovery health, Well-being, Work break conditions  Recovery during work Teamwork, Home-work interference | General hospital with three locations in the Eastern part of Netherlands |
| Niks et al. 2018^17^ | n=111 | Job demands, Job resource, Recovery health, Well-being, Work break conditions  Recovery during work Teamwork, Home-work interference | Multi-located Dutch general hospital |
| Ruitenberg et al. 2015^18^ | n= 32/30 | Impaired work functioning, distress, work-related fatigue, posttraumatic stress & work ability. | Academic Medical Center in the Netherlands |
| Ruitenberg et al. 2016^19^ | NA | NA | Academic Medical Center in the Netherlands |
| Schneider et al. 2019^20^ | n= 149 | Emotional exhaustion Depersonalization | Tertiary referral hospital |
| Shanafelt et al. 2014^21^ | n= 1150 | Behavioral intentions, Physician Well-being index | nr |
| Uchiyama et al. 2013^22^ | n= 401 | Mental health Psychosocial work environment | Two private, medium-sized general hospitals in Japan |
| Weiner et al. 2020^23^ | NA | Perceived stress Depression Post-traumatic stress symptoms Resilience Insomnia Work-related rumination Credibility Satisfaction. | Six hospitals of the East region of France |

1. Arrigoni C, Caruso R, Campanella F, Berzolari FG, Miazza D, Pelissero G. Investigating burnout situations, nurses' stress perception and effect of a post-graduate education program in health care organizations of northern Italy: a multicenter study. *G Ital Med Lav Ergon.* 2015;37(1):39-45.

2. Blake H, Bermingham F, Johnson G, Tabner A. Mitigating the Psychological Impact of COVID-19 on Healthcare Workers: A Digital Learning Package. *Int J Environ Res Public Health.* 2020;17(9).

3. d'Ettorre G, Greco M. Healthcare Work and Organizational Interventions to Prevent Work-related Stress in Brindisi, Italy. *Saf Health Work.* 2015;6(1):35-38.

4. Di Tecco C, Nielsen K, Ghelli M, et al. Improving Working Conditions and Job Satisfaction in Healthcare: A Study Concept Design on a Participatory Organizational Level Intervention in Psychosocial Risks Management. *Int J Environ Res Public Health.* 2020;17(10).

5. Ericson-Lidman E, Ahlin J. Assessments of stress of conscience, perceptions of conscience, burnout, and social support before and after implementation of a participatory action-research-based intervention. *Clinical Nursing Research.* 2017;26(2):205-223.

6. Gartner FR, Ketelaar SM, Smeets O, et al. The Mental Vitality @ Work study: design of a randomized controlled trial on the effect of a workers' health surveillance mental module for nurses and allied health professionals. *BMC Public Health.* 2011;11:290.

7. Gartner FR, Nieuwenhuijsen K, Ketelaar SM, van Dijk FJ, Sluiter JK. The Mental Vitality @ Work Study: Effectiveness of a mental module for workers' health surveillance for nurses and allied health care professionals on their help-seeking behavior. *Journal of Occupational and Environmental Medicine.* 2013;55(10):1219-1229.

8. Havermans BM, Boot CR, Brouwers EP, et al. Effectiveness of a digital platform-based implementation strategy to prevent work stress in a healthcare organization: a 12-month follow-up controlled trial. *Scand J Work Environ Health.* 2018;44(6):613-621.

9. Isaksson Ro KE, Tyssen R, Hoffart A, Sexton H, Aasland OG, Gude T. A three-year cohort study of the relationships between coping, job stress and burnout after a counselling intervention for help-seeking physicians. *BMC Public Health.* 2010;10:213.

10. Ketelaar SM, Gartner FR, Bolier L, Smeets O, Nieuwenhuijsen K, Sluiter JK. Mental Vitality @ Work-A workers' health surveillance mental module for nurses and allied health care professionals: Process evaluation of a randomized controlled trial. *Journal of Occupational and Environmental Medicine.* 2013;55(5):563-571.

11. Ketelaar SM, Nieuwenhuijsen K, Gartner FR, Bolier L, Smeets O, Sluiter JK. Effect of an E-mental health approach to workers' health surveillance versus control group on work functioning of hospital employees: a cluster-RCT. *PLoS ONE.* 2013;8(9):e72546.

12. Ketelaar SM, Nieuwenhuijsen K, Bolier L, Smeets O, Sluiter JK. Improving work functioning and mental health of health care employees using an e-mental health approach to workers' health surveillance: pretest-posttest study. *Saf Health Work.* 2014;5(4):216-221.

13. Ketelaar SM, Nieuwenhuijsen K, Gartner FR, Bolier L, Smeets O, Sluiter JK. Mental Vitality @ Work: The effectiveness of a mental module for workers' health surveillance for nurses and allied health professionals, comparing two approaches in a cluster-randomised controlled trial. *Int Arch Occup Environ Health.* 2014;87(5):527-538.

14. Le Blanc PM, Hox JJ, Schaufeli WB, Taris TW, Peeters MC. Take care! The evaluation of a team-based burnout intervention program for oncology care providers. *J Appl Psychol.* 2007;92(1):213-227.

15. Moll S, Patten SB, Stuart H, Kirsh B, MacDermid JC. Beyond silence: protocol for a randomized parallel-group trial comparing two approaches to workplace mental health education for healthcare employees. *BMC Med Educ.* 2015;15:78.

16. Niks I, de Jonge J, Gevers J, Houtman I. Work Stress Interventions in Hospital Care: Effectiveness of the DISCovery Method. *Int J Environ Res Public Health.* 2018;15(2).

17. Niks IM, de Jonge J, Gevers JM, Houtman IL. Design of the DISCovery project: tailored work-oriented interventions to improve employee health, well-being, and performance-related outcomes in hospital care. *BMC Health Serv Res.* 2013;13:66.

18. Ruitenburg MM, Frings-Dresen MH, Sluiter JK. How to Define the Content of a Job-Specific Worker's Health Surveillance for Hospital Physicians? *Saf Health Work.* 2016;7(1):18-31.

19. Ruitenburg MM, Plat MC, Frings-Dresen MH, Sluiter JK. Feasibility and acceptability of a workers' health surveillance program for hospital physicians. *Int J Occup Med Environ Health.* 2015;28(4):731-739.

20. Schneider A, Wehler M, Weigl M. Effects of work conditions on provider mental well-being and quality of care: a mixed-methods intervention study in the emergency department. *BMC Emerg Med.* 2019;19(1):1.

21. Shanafelt TD, Kaups KL, Nelson H, et al. An interactive individualized intervention to promote behavioral change to increase personal well-being in US surgeons. *Ann Surg.* 2014;259(1):82-88.

22. Uchiyama A, Odagiri Y, Ohya Y, Takamiya T, Inoue S, Shimomitsu T. Effect on mental health of a participatory intervention to improve psychosocial work environment: a cluster randomized controlled trial among nurses. *J Occup Health.* 2013;55(3):173-183.

23. Weiner L, Berna F, Nourry N, Severac F, Vidailhet P, Mengin AC. Efficacy of an online cognitive behavioral therapy program developed for healthcare workers during the COVID-19 pandemic: the REduction of STress (REST) study protocol for a randomized controlled trial. *Trials.* 2020;21(1):870.
