# Supplementary material for: Risk communication about work-related stress disorders in healthcare workers: a scoping review
Source: Int Arch Occup Environ Health. 2022 Mar 16;95(6):1195–208. doi: 10.1007/s00420-022-01851-x (PMC8923828; doi:10.1007/s00420-022-01851-x)
Supplement: Supplementary file 2 — Supplementary file2 (DOCX 53 kb) [file 420_2022_1851_MOESM2_ESM.docx]

Appendix 2.

|  | Elements of risk communication | | | | Goals of risk communication | | |
| --- | --- | --- | --- | --- | --- | --- | --- |
|  | Risk perception | Communication about: early stress | Communication about: risk factors | Communication about: prevention | informing | Stimulating informed decision making | Motivating taking action |
| Arrigoni et al. 2015^1^ | Two-way dialogue between the researchers and the nurses, which shaped the content of the educational plan. The exercises in the educational plan were based on real cases brought in by the nurses themselves | (early) symptoms of the level of perceived stress | Stressful work situations | Coping with stress on problem solving, social support, emotional distress and problem avoidance. | By the means of an educational plan | The educational  plan had to improve the perception of being able to better  manage the stressful situations | Using best strategies to  identify the problem and to address attention on peer  group, which was an essential resource. The peer group relationship could have a preventive role; however in cases  of full-blown BO, a specialist intervention is required. |
| Blake et al. 2020^2^ | - | Key Symptoms of Sustained Stress,  Risk Factors for Psychological Ill-Health | The Impact of Workplace on Psychological Wellbeing,  how to Improve, the Working Environment  Working under Pressure in a Team | Telephone Helplines  Caring for Doctors Caring for Patients  Downloadable Wellbeing Posters  Mental Health Guidance  Wellbeing and Resilience Guidance  Stress and Resilience at Work  Directory of Support | Enable users to be better informed about psychological issues and impacts during and after a pandemic | Encourage help-seeking behaviour | Providing evidence-based information, support and signposting for users |
| D'ettore et al. 2014^3^ | - | - | Sentinel events,  Work content factors,  Work context factors | Results of the assessment were fed back and prevention was based on this information | By means of a training | The training took place through lectures in two meetings with each participant about the organizational changes necessary to prevent WRS After implementing the improvement organizational actions | Corrective actions to prevent work-related stressdis were based on the results of the assessment |
| Di Tecco et al. 2020^4^ | Risk assessment, by surveying  employees’ perceptions of psychosocial risk factors | - | Communication about risk factors included working conditions and job satisfaction | Corrective/improvement action priorities based on the assessment results | Steer-group was informed about risk factors | Plan for actions was discussed | To change their work behaviors that actions require |
| Ericson-Lidman et al 2017^5^ | Participants identified, prioritized, and brainstormed about a chosen situation that generated troubled conscience, reviewed their knowledge about the situations and defined needs for gaining knowledge | Behavioral symptoms | Deficient team work, nonfunctional mealtime schedule | With the knowledge thus gained as a base, they decided on meaningful and feasible actions to be undertaken, and subsequently evaluated them | During participatory action research sessions knowledge about what caused troubled conscious was discussed | Knowledge to learn to constructively deal with troubled conscience | Deal with troubled conscience |
| Gartner et al. 2011 2013^6,7^  Ketelaar et al. 2013 2014^8,9^ | - | Mental health complaints | Work functioning impairments | The occupational physician communicated reduction of health complaints and for the improvement of work functioning and the prevention of incidents at work and discussing | Information provided by the occupational physician & results of the survey were fed back | Advice is given by the occupational physician about prevention, HCWs are provided with information to tackle the problems | Reduce mental health complaints and improve work functioning impairments |
| Havermans et al. 2021^10^ | - | Determinants for stress | Determinants for stress | Preventive interventions are chosen via the platform | Awareness raising acknowledgment of the fact that  management of work stress is important | By means of the intervention HCWs are informed and stimulated to take action | By implementing an intervention to reduce work-related stress |
| Isaksson et al. 2010^11^ | work-related and private contextual factors contributing to stress according to HCWs themselves | Emotional exhaustion,  job stress | - | Recommendation from the physician how to deal with theirs needs | By the physician during a consultation | By means of the intervention HCWs are informed and stimulated to take action | Stress reduction or obtaining treatment, such as psychotherapy |
| Ketelaar et al. 2013 2014^12,13^ | - | mental health complaints | work functioning impairments | Online tailored advice, consisting of an invitation to follow an EMH intervention and (if applicable) the receipt of an onscreen educational leaflet with advice per subscale on how to improve work functioning | Digital information | By means of the intervention HCWs are informed and stimulated to take action | HCWs are motivated to take action towards their (mental) health if they are at risk |
| Le Blanc et al. 2007^14^ | Discussing the HCWs perception of main sources of job stress. This was performed by interviews, where the HCWs local context was taken into account | - | Working mechanisms of job stress  social support network and balancing job-related investments | A plan to take action was discussed with the HCWs | Information about risk factors was communicated | By means of the intervention HCWs are informed and stimulated to take action | Designed to diminish anxiety and cope with the most important stressors in their work situation |
| Moll et al. 2015^15^ | - | Mental health | - | Teach skills to reach out to seek/offer help | Information sharing | By means of the intervention HCWs are informed and stimulated to take action | Reduce stigma and increase confidence and skill in reaching out to seek/offer help |
| Niks et al. 2013, 2018^16,17^ | - | - | Job demands,  job resources | HCWs discussed potential solutions and defined a plan with concrete measures | By means of a PAR information about job demands and job resources was discussed | By means of the intervention HCWs are informed and stimulated to take action | Implementing an organizational-intervention to reduce work-related stress |
| Ruitenberg et al. 2015, 2016^18,19^ | - | Psychological health complaints and posttraumatic stress complaints | Risk factors such as aggression, violence, work ability | Information and advice or an offer of a targeted intervention provided by the occupational physician | Information is given by the occupational physician | After the consult with the occupational physician | To detect and prevent work-related health complaints in early stages and to improve work ability of hospital physicians |
| Schneider et al. 2019^20^ | - | Work stress. Emotional exhaustion, depersonalization, depressive symptoms, job satisfaction | Patient  stressors  job participation opportunities  work overload  Organizational factors consisted of  personnel resources  information  job insecurity  social  and supervisor  feedback | Classified problematic work conditions according to their practical importance and potential for change and formed an agenda of issues for improvement for subsequent meetings. | Information was provided during the meetings | By means of the intervention HCWs are informed and stimulated to take action | HCWs to take action towards emergency department factors that influence their health |
| Shanafelt et al. 2014^21^ | - | Career satisfaction, meaning in work | Fatigue, risk of suicidal ideation, risk degree of distress | The feedback included the phone number for the National Suicide Prevention hotline. | Via the survey information about the average of others HCWs was provided | By means of the intervention HCWs are informed and stimulated to take action | If at risk HCWs were motivated to take action towards their mental health |
| Uchiyama et al. 2013^22^ | - | Mental health | Psychosocial risk factors | HCW themselves came up with solutions | By participating with the participatory action research | By means of the intervention HCWs are informed and stimulated to take action | action planning to improve the work environment |
| Weiner et al. 2020^23^ | - | The psychological mechanisms of stress | Work system factors | Useful behavior during stressful situations | Information is provided via the ‘my health too’ web-based intervention | By means of the intervention HCWs are informed and stimulated to take action | Increasing resilience to cope with stress |

1. Arrigoni C, Caruso R, Campanella F, Berzolari FG, Miazza D, Pelissero G. Investigating burnout situations, nurses' stress perception and effect of a post-graduate education program in health care organizations of northern Italy: a multicenter study. *G Ital Med Lav Ergon.* 2015;37(1):39-45.

2. Blake H, Bermingham F, Johnson G, Tabner A. Mitigating the Psychological Impact of COVID-19 on Healthcare Workers: A Digital Learning Package. *Int J Environ Res Public Health.* 2020;17(9).

3. d'Ettorre G, Greco M. Healthcare Work and Organizational Interventions to Prevent Work-related Stress in Brindisi, Italy. *Saf Health Work.* 2015;6(1):35-38.

4. Di Tecco C, Nielsen K, Ghelli M, et al. Improving Working Conditions and Job Satisfaction in Healthcare: A Study Concept Design on a Participatory Organizational Level Intervention in Psychosocial Risks Management. *Int J Environ Res Public Health.* 2020;17(10).

5. Ericson-Lidman E, Ahlin J. Assessments of stress of conscience, perceptions of conscience, burnout, and social support before and after implementation of a participatory action-research-based intervention. *Clinical Nursing Research.* 2017;26(2):205-223.

6. Gartner FR, Ketelaar SM, Smeets O, et al. The Mental Vitality @ Work study: design of a randomized controlled trial on the effect of a workers' health surveillance mental module for nurses and allied health professionals. *BMC Public Health.* 2011;11:290.

7. Gartner FR, Nieuwenhuijsen K, Ketelaar SM, van Dijk FJ, Sluiter JK. The Mental Vitality @ Work Study: Effectiveness of a mental module for workers' health surveillance for nurses and allied health care professionals on their help-seeking behavior. *Journal of Occupational and Environmental Medicine.* 2013;55(10):1219-1229.

8. Ketelaar SM, Gartner FR, Bolier L, Smeets O, Nieuwenhuijsen K, Sluiter JK. Mental Vitality @ Work-A workers' health surveillance mental module for nurses and allied health care professionals: Process evaluation of a randomized controlled trial. *Journal of Occupational and Environmental Medicine.* 2013;55(5):563-571.

9. Ketelaar SM, Nieuwenhuijsen K, Gartner FR, Bolier L, Smeets O, Sluiter JK. Mental Vitality @ Work: The effectiveness of a mental module for workers' health surveillance for nurses and allied health professionals, comparing two approaches in a cluster-randomised controlled trial. *Int Arch Occup Environ Health.* 2014;87(5):527-538.

10. Havermans BM, Boot CR, Brouwers EP, et al. Effectiveness of a digital platform-based implementation strategy to prevent work stress in a healthcare organization: a 12-month follow-up controlled trial. *Scand J Work Environ Health.* 2018;44(6):613-621.

11. Isaksson Ro KE, Tyssen R, Hoffart A, Sexton H, Aasland OG, Gude T. A three-year cohort study of the relationships between coping, job stress and burnout after a counselling intervention for help-seeking physicians. *BMC Public Health.* 2010;10:213.

12. Ketelaar SM, Nieuwenhuijsen K, Bolier L, Smeets O, Sluiter JK. Improving work functioning and mental health of health care employees using an e-mental health approach to workers' health surveillance: pretest-posttest study. *Saf Health Work.* 2014;5(4):216-221.

13. Ketelaar SM, Nieuwenhuijsen K, Gartner FR, Bolier L, Smeets O, Sluiter JK. Effect of an E-mental health approach to workers' health surveillance versus control group on work functioning of hospital employees: a cluster-RCT. *PLoS ONE.* 2013;8(9):e72546.

14. Le Blanc PM, Hox JJ, Schaufeli WB, Taris TW, Peeters MC. Take care! The evaluation of a team-based burnout intervention program for oncology care providers. *J Appl Psychol.* 2007;92(1):213-227.

15. Moll S, Patten SB, Stuart H, Kirsh B, MacDermid JC. Beyond silence: protocol for a randomized parallel-group trial comparing two approaches to workplace mental health education for healthcare employees. *BMC Med Educ.* 2015;15:78.

16. Niks I, de Jonge J, Gevers J, Houtman I. Work Stress Interventions in Hospital Care: Effectiveness of the DISCovery Method. *Int J Environ Res Public Health.* 2018;15(2).

17. Niks IM, de Jonge J, Gevers JM, Houtman IL. Design of the DISCovery project: tailored work-oriented interventions to improve employee health, well-being, and performance-related outcomes in hospital care. *BMC Health Serv Res.* 2013;13:66.

18. Ruitenburg MM, Frings-Dresen MH, Sluiter JK. How to Define the Content of a Job-Specific Worker's Health Surveillance for Hospital Physicians? *Saf Health Work.* 2016;7(1):18-31.

19. Ruitenburg MM, Plat MC, Frings-Dresen MH, Sluiter JK. Feasibility and acceptability of a workers' health surveillance program for hospital physicians. *Int J Occup Med Environ Health.* 2015;28(4):731-739.

20. Schneider A, Wehler M, Weigl M. Effects of work conditions on provider mental well-being and quality of care: a mixed-methods intervention study in the emergency department. *BMC Emerg Med.* 2019;19(1):1.

21. Shanafelt TD, Kaups KL, Nelson H, et al. An interactive individualized intervention to promote behavioral change to increase personal well-being in US surgeons. *Ann Surg.* 2014;259(1):82-88.

22. Uchiyama A, Odagiri Y, Ohya Y, Takamiya T, Inoue S, Shimomitsu T. Effect on mental health of a participatory intervention to improve psychosocial work environment: a cluster randomized controlled trial among nurses. *J Occup Health.* 2013;55(3):173-183.

23. Weiner L, Berna F, Nourry N, Severac F, Vidailhet P, Mengin AC. Efficacy of an online cognitive behavioral therapy program developed for healthcare workers during the COVID-19 pandemic: the REduction of STress (REST) study protocol for a randomized controlled trial. *Trials.* 2020;21(1):870.
