# Supplementary material for: Risk communication about work-related stress disorders in healthcare workers: a scoping review
Source: Int Arch Occup Environ Health. 2022 Mar 16;95(6):1195–208. doi: 10.1007/s00420-022-01851-x (PMC8923828; doi:10.1007/s00420-022-01851-x)
Supplement: Supplementary file 3 — Supplementary file3 (DOCX 17 kb) [file 420_2022_1851_MOESM3_ESM.docx]

Appendix 3.

|  | **Ovid MEDLINE(R) ALL <1946 to December 14, 2020> Search date: 15 December 2020** |  |
| --- | --- | --- |
| **#** | **Searches** | **Hits** |
| 1 | exp occupational stress/ | 14416 |
| 2 | (((job or work or occupation*) adj2 stress) or compassion fatigue or burnout or burn out or ((strain? or demand*) adj2 (work or job or profession?))).ab,kf,ti. | 26970 |
| 3 | 1 or 2 [occupational stress precise] | 32301 |
| 4 | (work abil* or work participa* or work functioning or "functioning at work").ab,kf,ti. [occupational stress sensitive] | 2965 |
| 5 | exp employment/ or exp work/ or "rehabilitation, vocational"/ or occupational health/ or occupational groups/ or sick leave/ or workers' compensation/ | 186086 |
| 6 | (paid work or worker? or vocational or occupation* or sick leave or absenteeism or presenteeism or employment or employee? or sickness absence or job? or work place or "return to work" or work related or work disabil* or work product* or work limit* or work instabili* or work performance or work capacit or work evaluat* or work direct* or working populat* or workplace).ab,kf,ti. | 478435 |
| 7 | 4 or 5 [work] | 187480 |
| 8 | (mental or psych*).mp. | 2260692 |
| 9 | (stress or emotion*).mp. | 1141103 |
| 10 | (anxiety or depression or ((heart or coronary or cardio*) adj2 (disease? or disorder?))).mp. [outcomes] | 1237815 |
| 11 | or/8-10 | 3868922 |
| 12 | 7 and 11 [occupational stress sensitive] | 65289 |
| 13 | 3 or 12 [occupational stress complete] | 88144 |
| 14 | (hospital? or healthcare organi?ation or icu or picu or nursing homes or eol facilit* or "end of life facilit*").ab,kf,ti. | 1233499 |
| 15 | (care professional? or healthcare professional? or elderly care or nurse? or healthcare worker? or care worker? or physician? or doctor?).mp. | 1021638 |
| 16 | 14 or 15 [healthcare workers specific] | 2089752 |
| 17 | (caregiver? or care provider?).mp. | 136691 |
| 18 | (public health or environmental medicine).in,mp. | 901915 |
| 19 | (public or environmental).jw. | 526207 |
| 20 | (educator? or police).mp. | 46163 |
| 21 | or/16-20 [healthcare workers sensitive] | 3365458 |
| 22 | (risk? and (perceived or perception or communicat*)).ab,kf,ti. | 81007 |
| 23 | organi?ational justice.ab,kf,ti. [very specific risk factor] | 343 |
| 24 | (decision latitude or decision authority or skill discretion or social support or effort reward).ab,kf,ti. [sensitive risk factors] | 42467 |
| 25 | ((sought or seek*) adj2 (help or aid)).ab,kf,ti. | 12330 |
| 26 | (prevent* adj2 (harm* or work or stress)).ab,kf,ti. | 9713 |
| 27 | (autonom* or bullying or bullied or cyberbullying).ab,hw,kf,ti. [risk factors] | 189030 |
| 28 | ((health surveillance adj2 (employee? or worker?)) or (interview* adj5 (employee? or worker?))).ab,kf,ti. | 3386 |
| 29 | or/22-28 [risk communication] | 331025 |
| 30 | and/13,21,29 | 5961 |
| 31 | (stress check program or "vital@work" or (vital* adj2 work) or (stress prevention adj2 work) or "prevention at work" or "vital at work" or "vitality at work").mp. [E - relevant studies] | 174 |
| 32 | 30 or 31 | 6115 |
| 33 | (family characteristics or mothers or infancy or newborn? or drinking or pregnanc* or cardiovascular disease or cardiovacular disorder or hospitali?ation or tobacco or sedentary behavior or obesity or obese or body mass index or family relation?).ab,kf,ti. [VOS green] | 1617414 |
| 34 | exp musculoskeletal diseases/ or schools/ or low back pain/ or physical exertion/ | 1205701 |
| 35 | (musculoskeletal or (school? not ((public health or medic*) adj2 school?)) or low back pain or physical exertion).ab,kf,ti. | 311702 |
| 36 | 34 or 35 [VOS blue] | 1440020 |
| 37 | 32 not (33 or 36) | 5145 |
|  |  |  |
|  |  |  |
|  | **Ovid APA PsycInfo <1806 to December Week 1 2020> Search date: 15 December 2020** |  |
| **#** | **Searches** | **Hits** |
| 1 | exp occupational stress/ | 22200 |
| 2 | (((job or work or occupation*) adj2 stress) or compassion fatigue or burnout or burn out or ((strain? or demand*) adj2 (work or job or profession?))).ab,id,ti. | 27446 |
| 3 | 1 or 2 | 32862 |
| 4 | exp health personnel/ | 165053 |
| 5 | (hospital? or healthcare organi?ation or icu or picu or nursing homes or eol facilit* or "end of life facilit*").ab,id,ti. | 134860 |
| 6 | (care professional? or healthcare professional? or elderly care or nurse? or healthcare worker? or care worker? or physician? or doctor?).mp. | 166892 |
| 7 | (caregiver? or care provider?).mp. | 79573 |
| 8 | (public health or environmental medicine).in,mp. | 162495 |
| 9 | (public or environmental).jx. | 31965 |
| 10 | (educator? or police).mp. | 78685 |
| 11 | or/4-10 [healthcare workers] | 618327 |
| 12 | preventive mental health services/ | 2429 |
| 13 | communication/ and risk.hw. | 841 |
| 14 | (risk? and (perceived or perception or communicat*)).ab,id,ti. | 48706 |
| 15 | organi?ational justice.ab,id,ti. [very specific risk factor] | 1628 |
| 16 | (decision latitude or decision authority or skill discretion or social support or effort reward).ab,id,ti. [sensitive risk factors] | 51589 |
| 17 | ((sought or seek*) adj2 (help or aid)).ab,id,ti. | 15190 |
| 18 | (prevent* adj2 (harm* or work or stress)).ab,id,ti. | 3688 |
| 19 | (autonom* or bullying or bullied or cyberbullying).ab,hw,id,ti. [risk factors] | 73825 |
| 20 | ((health surveillance adj2 (employee? or worker?)) or (interview* adj5 (employee? or worker?))).ab,id,ti. | 3545 |
| 21 | or/12-20 [risk communication] | 193246 |
| 22 | and/3,11,21 | 2146 |
| 23 | (family characteristics or mothers or infancy or newborn? or drinking or pregnanc* or cardiovascular disease or cardiovacular disorder or hospitali?ation or tobacco or sedentary behavior or obesity or obese or body mass index or family relation?).ab,id,ti. [VOS green] | 298827 |
| 24 | (musculoskeletal or (school? not ((public health or medic*) adj2 school?)) or low back pain or physical exertion).ab,id,ti. [VOS blue] | 399203 |
| 25 | 23 or 24 | 672487 |
| 26 | 22 not 25 | 1876 |
|  |  |  |
|  |  |  |
|  | **Web of Science Indexes=SCI-EXPANDED, SSCI, A&HCI, ESCI Timespan=All years Search date: 15 December 2020** |  |
| **#** | **Searches** | **Hits** |
| 1 | TS=(((job or work or occupation*) near/1 stress) or "compassion fatigue" or burnout or "burn out" or ((strain? or demand*) near/1 (work or job or profession?))) | 50235 |
| 2 | TS=(("work abil*" or "work participa*" or "work functioning" or "functioning at work") and stress) | 497 |
| 3 | #1 or #2 | 50484 |
| 4 | TS=(hospital? or "healthcare organization" or icu or picu or "nursing homes" or "eol facilit*" or "end of life facilit*") | 265571 |
| 5 | TS=("care professional?" or "healthcare professional?" or "elderly care" or nurse? or "healthcare worker?" or "care worker?" or physician? or doctor?) | 431751 |
| 6 | TS=(caregiver? or "care giver?" or "care provider?") | 103901 |
| 7 | TS=(educator? or police) | 100823 |
| 8 | #4 or #5 or #6 or #7 | 830518 |
| 9 | TS=(risk? and (perceived or perception or communicat*)) | 34040 |
| 10 | TS="organizational justice" | 2936 |
| 11 | TS=("decision latitude" or "decision authority" or "skill discretion" or "social support" or "effort reward") | 73865 |
| 12 | TS=((sought or seek*) near/1 (help or aid)) | 17906 |
| 13 | TS=(prevent* near/1 (harm* or work or stress)) | 11416 |
| 14 | TS=(autonom* or bullying or bullied or cyberbullying) | 253826 |
| 15 | TS=(("health surveillance" near/1 (employee? or worker?)) or (interview* near/4 (employee? or worker?))) | 4827 |
| 16 | #9 or #10 or #11 or #12 or #13 or #14 or #15 | 392650 |
| 17 | #3 and #8 and #16 | 2486 |
| 18 | TS=("family characteristics" or mothers or infancy or newborn? or drinking or pregnanc* or "cardiovascular disease" or "cardiovacular disorder" or hospitalization or tobacco or "sedentary behavior" or obesity or obese or "body mass index" or "family relation?") | 1820154 |
| 19 | TS=(musculoskeletal or (school? not (("public health" or medic*) near/1 school?)) or "low back pain" or "physical exertion") | 255643 |
| 20 | #17 not (#18 or #19) | 2237 |
| 21 | TS=("stress check program" or "vital@work" or (vital* near/1 work) or ("stress prevention" near/1 work) or "prevention at work" or "vital at work" or "vitality at work") | 339 |
| 22 | #20 or #21 | 2569 |
